# A door opener to physical capability and social community: children’s and parents’ experiences of physical activity on prescription by the Swedish school health service

**DOI:** 10.1080/17482631.2026.2731530

**Published:** 2026-09-09

**Authors:** Emelie Wiklund, Maria Wiklund, Jenny Vikman, Susanna Hedenborg

**Affiliations:** a Department of Sports Sciences, Malmö University, Malmö, Sweden; b Physiotherapy unit, Department of Community Medicine and Rehabilitation, Umeå University, Umeå, Sweden

**Keywords:** Children, health promotion, health resources, parents, physical activity, physical activity on prescription, salutogenesis, school health services

## Abstract

**Purpose:**

Physical Activity on Prescription (PAP) is increasingly implemented within Swedish school health services, yet little is known about how it is experienced in educational settings. Existing research on school-based physical activity interventions has predominantly focused on medical outcomes, often overlooking contextual, sociocultural, and experiential dimensions. This study aims to explore how children and their parents experience receiving PAP in Swedish schools, using a salutogenic framework focused on health resources and sociocultural integration.

**Methods:**

An inductive qualitative design was employed. Individual interviews were conducted with seven children aged 9–17 years and seven parents. Data were analyzed using qualitative content analysis.

**Results:**

PAP was experienced as a “door opener” to physical capability and social community on participants’ own terms. Tailored and accessible activities, embedded in supportive social environments, facilitated engagement in meaningful movement. These experiences were conceptualized and interpreted through a salutogenic lens as mobilization of health resources, including enhanced well-being, social connectedness, increased motivation, and facilitated engagement in enjoyable movement.

**Conclusions:**

The findings highlight the importance of resource-oriented and context-sensitive approaches in school-based health interventions. PAP may support children’s well-being beyond biomedical outcomes by enabling participation in meaningful and socially embedded physical activity when adapted to individual needs.

## Introduction

Physical activity habits that promote health should be established during childhood (Lockenwitz Petersen et al., 2020). Children’s perspectives are central when developing physical activity promotion (Emm-Collison et al., 2022). Acknowledging children as active agents rather than passive recipients (James & Prout, 2014) is, thus, essential for developing effective and equitable approaches to physical activity promotion (Larsson et al., 2018). Also, parents play a crucial role in promoting physical activity habits by providing opportunities and serving as role models (Maric et al., 2020; Mitchell et al., 2012), whereas their lack of support can hinder physical activity (Noonan et al., 2016). Moreover, schools are important environments for promoting physical activity (Neil-Sztramko et al., 2021; Wang et al., 2025; Yuksel et al., 2020).

Therefore, shaping children’s movements requires both child participation, parental support and a health-promoting school environment; integrating these supportive influences can achieve sustainable improvements in children’s movement behaviours (Messing et al., 2019; Rhodes and Guerrero, 2020).

In schools, physical activity is promoted through physical education, recess activities, and extracurricular physical activities (Jägerbrink et al., 2022; Svanelöv, 2024). However, these efforts often have limited long-term impact and do not reach all children (Larsson & Thedin Jakobsson, 2023; Yuksel et al., 2020). For instance, recess physical activity can be hindered by power hierarchies, social barriers, and social codes (Svanelöv, 2024), and extracurricular physical activities struggle to engage inactive children outside of school (Jägerbrink et al., 2022).

In some Swedish schools, the school health service (SHS) uses physical activity on prescription (PAP) to engage children who need additional physical activity. PAP involves structured, individualised prescriptions for physical activity (Kallings et al., 2021). It was initially developed within health care for adults with specific diagnoses to increase physical activity levels (Onerup et al., 2019). For children, PAP is based on non-medical assessments by licensed healthcare professionals in consultation with the child and their parents. Some municipalities have developed co-organised PAP, providing a supportive network of adults during PAP. PAP activities are tailored to each child and include everyday exercises, organised activities, or subsidised options such as swimming and gym workouts (Wiklund et al., 2025).

Research on PAP for children in schools is limited. Existing research shows that school nurses view PAP as a useful tool for promoting joyful health and motivating physical activity, although it requires careful tailoring to each child’s needs and social conditions (Wiklund et al., 2025). However, there is still limited knowledge regarding how PAP is experienced by children and their parents in school contexts.

Implementing established methods in new settings (Skivington et al., 2021), like PAP in schools, requires understanding the experiences of the users, which in this context include children and their parents. Although PAP has been used in several Swedish municipalities, there is limited knowledge about its application in schools and scarce insights from children and parents. Therefore, this study aimed to contribute to knowledge on PAP by exploring children’s and their parents’ experiences of receiving PAP in Swedish schools.

### Salutogenic perspective as an analytical lens

In this study, we use Aaron Antonovsky’s salutogenic theory (1979) on ‘sense of coherence’ as our analytical lens, focusing on ‘health resources’. Examples of health resources include personal well-being, social relations, and meaningful activities (Geidne & Quennerstedt, 2021). Mobilising such resources helps individuals perceive life as comprehensible, manageable, and meaningful—the three components of sense of coherence (Eriksson & Lindström, 2006).

According to Larsson and Thedin Jakobsson (2023), school-based physical activity interventions often adopt a positivistic and pathogenic medical perspective, aiming to ‘change individuals and their behaviour rather than school environments and their workings’. This highlights a gap between medical and social sciences (Kretchmar, 2008). Although PAP was initially developed in a medical context (Kallings et al., 2021), it is not strictly linked to medical diagnoses for children. Therefore, a salutogenic perspective, that is, a health-promoting perspective using a broad and socially informed definition of health, is useful for exploring PAP. By understanding PAP through this lens, we seek to address the research gap and gain insights into children’s and parents’ experiences of PAP. Such insights are crucial for designing interventions that align with the real needs and social conditions of children, thereby fostering a supportive environment for long-term physical activity.

## Methods

### Study design and context

This study used an inductive qualitative approach (following Graneheim et al., 2017) with a salutogenic theoretical lens (Antonovsky, 1979) to analyse individual interviews with children and parents who have experience with PAP in a Swedish school setting. Although Antonovsky originally operationalized sense of coherence through a quantitative scale, the concept has also been applied in qualitative research to explore how individuals experience and mobilise health resources in everyday life (Geidne & Quennerstedt, 2021). PAP was administrated by school nurses in school health service (SHS).

### Procedure and participants

The inclusion criteria were (1) children aged 9–18 years who had an ongoing or completed PAP during the last year, and (2) parents to a child aged 9 –18 years who had an ongoing or completed PAP during the last year. Participants were recruited from schools (*n* = 5) where PAP was implemented. The schools represented three different municipalities, including two municipalities in southern Sweden (small and medium size) and one in northern Sweden (medium size), and encompassed varied geographic areas (rural and urban) and different socioeconomic indexes. The selection of schools and municipalities was strategic in that PAP had been implemented in these settings, while recruitment within participating schools aimed to include all children and parents who met the inclusion criteria and were identified by the school nurses.

Recruitment took place from September 2022 to May 2024. Initial contact with information about the study was made via email to SHS managers in all three municipalities. The SHS managers subsequently informed school nurses (in person or via e-mail) about the study and their potential recruitment role and passed their contact details to the first author. Approximately 30 school nurses were approached, of whom approximately half agreed to assist with recruitment. The school nurses who agreed to assist received standardised information about the study and acted as key gatekeepers—informing children and parents about the study in person, by letter, or by telephone. The school nurses approached children and parents of children who had received PAP during the previous year and asked whether they were interested in participating in the study. Those who were interested were provided with further information and their contact details were passed to the authors. The exact number of children and parents approached was not systematically recorded by the school nurses and could therefore not be reported. Consequently, the numbers declining participation or not responding could not be determined. No children or parents who expressed an interest in participating were subsequently excluded from the study. Reminders about the study were sent to the school nurses before each new term. During data collection, five additional municipalities began implementing the PAP programme and were contacted using the same approach. One of these five municipalities ultimately contributed participants, namely three children and one parent. Thus, eight municipalities were contacted during the recruitment period. The final participants were recruited from five schools across three municipalities. The intention was to recruit all children and parents who met the inclusion criteria and were identified through the participating schools. Recruitment was concluded after seven children and seven parents had participated, as the research team considered the interview material sufficiently information-rich to address the study aim.

A total of 14 participants were included in the study: seven children (three girls and four boys) and seven parents (four women and three men). The ages of the children ranged from 9 to 17 years. Detailed participant characteristics are presented in Table I. All participating parents were guardians of a child who also participated in the study. Two of the participating children had both their mother and father participating as parent participants. The participants were relatively homogeneous, but there was also heterogeneity. For instance, the country of birth and first language varied among the participants; most were born in Sweden and had Swedish as their first language, but there were also participants who were born in non-European countries and had Arabic as a first language. Some of the children had mental disorders (ADHD, autism, and chromosomal abnormality). Further, not all of the children had previous experience of physical activity in organised sports, whereas most of the parents indicated that they had previous experience with physical activity and some had also participated in sports clubs in childhood. The children had chosen different physical activities; the majority chose swimming, but some opted for individual activities (walking with pedometers or visiting playgrounds) and football in an organised club.

**Table I.** Detailed information about the participant .

Participant	Age	Gender	Born in Sweden	First language	PAP activity	PAP status	Interview mode
Child 1	12	Girl	Yes	Swedish	Walking with pedometers	Completed	Telephone
Child 2	13	Boy	No	Arabic	Football	Completed	Telephone
Child 3	9	Boy	Yes	Swedish	Swimming, playground visit	Completed	Live
Child 4	9	Boy	Yes	Arabic	Swimming	Ongoing	Live
Child 5	17	Girl	Yes	Swedish	Swimming	Ongoing	Zoom
Child 6	11	Boy	Yes	Swedish	Swimming	Completed	Zoom
Child 7	16	Girl	Yes	Swedish	Swimming	Completed	Zoom
Parent 1	45	Man	Yes	Swedish	Walking with pedometers	Completed	Telephone
Parent 2	43	Women	No	Thai	Walking with pedometers	Completed	Telephone
Parent 3	40	Women	No	Arabic	Football	Completed	Telephone
Parent 4	42	Man	Yes	Swedish	Swimming, playground visit	Completed	Live
Parent 5	40	Women	No	Arabic	Swimming	Ongoing	Live
Parent 6	47	Man	No	Arabic	Swimming	Ongoing	Zoom
Parent 7	37	Women	Yes	Swedish	Swimming	Completed	Zoom

### Data collection

The data was collected by the first author through semi-structured interviews (Moser & Korstjens, 2017). The participants were allowed to choose the time and place for the interview themselves. The interviews were conducted in person, by videoconferencing tools (Teams), or by mobile phone and were recorded. In general, the younger children and their parents chose in-person meetings, whereas older children and their parents chose digital options: either video calls via Teams or audio calls by mobile phone. The interviews lasted in mean of 40 minutes. Two interview guides were used, one for children and one for parents, to explore experiences related to three thematic areas: experiences of PAP, support from school and family, and views on the future development of PAP. An overview of the interview guides and the main question areas for children and parents is presented in Table II. The guides included open-ended questions within each thematic area and were complemented with follow-up questions based on the participants’ answers. The interview guide for the children was pilot tested with two children within the age range selected for the study and then ethically reviewed. Each interview with the children began with a neutral question: What do you like to do in your free time? In addition, background questions regarding including age, school grade, birthplace, first language, physical activity level, and experiences of physical activity were asked at the beginning of the interview with the children and parents.

**Table II.** Overview of the interview guides for children and parents.

Children	Parents
**Experiences of PAP** Experiences and perceptions of PAP, motivation, activities, range of activities, family involvement, follow-up, and physical activity after PAP	**Experiences of PAP** Experiences and perceptions of PAP, motivation, activities, range of activities, family involvement, follow-up, and changes in family lifestyle and physical activity
**Support from school and family** Support from school and family, physical activity at school, need for additional support, and social/environmental influences	**Support from school and family** Support from school and healthcare professionals, parental involvement, knowledge and support needs, barriers to following PAP, and long-term changes
**Future development of PAP** Preferences regarding activities, settings, support, social interaction, and continuation of PAP	**Future development of PAP** Preferences regarding support, activities and providers, and views on the future of PAP

As there were children who had mental and neuropsychiatric disabilities, the interview situation was adapted to the best interests of the child. The adaptations included using image support for movement and physical activity, providing an environment without distractions, and choosing the time and place for the interview based on the child's needs. The children were asked whether they wanted a parent to be present during the interview or not. All child interviews were conducted separately from the parent interviews, and the children were offered the opportunity to participate without a parent present. In five out of seven child interviews, a parent was present at the child’s own request and was able to support the child, particularly the younger children and children with additional needs. The questions were directed to the child, who provided the answers. The parent could occasionally supplement the child’s response or clarify the child’s answer, but the interviewer ensured that the child was given the opportunity to answer and express their own perspective. In the analysis, only the children’s responses were analysed; parental comments were not included as data from the children.

### Data analysis

The interviews were transcribed verbatim and analysed using qualitative content analysis with an inductive approach (Graneheim & Lundman, 2004). Each interview was first summarised to capture the ‘naive’ understanding and get a sense of the whole. Thereafter, meaning units were condensed and provided with codes, which interpreted the manifest content on a slightly higher level of abstraction (while remaining closely related to descriptive data). The codes were continuously compared to identify both differences and similarities. Differences and similarities between children’s and parents’ accounts were retained during the analysis and considered when interpreting the sub-themes and overarching theme, see example in Table III. for example. Codes with similar meanings were then brought together into sub-categories, which in turn were abstracted and interpreted into four sub-themes representing latent content. Finally, one theme was formulated that captured the latent content running as a ‘red thread’ through all the sub-categories and sub-themes (Graneheim & Lundman, 2004). Detailed overviews are presented in Table IV. Quotations were selected from the interview material to illustrate the identified theme and sub-themes and to provide examples of participants´ experiences. The selection was based on the analytical relevance and illustrative value of the excerpts rather that on an intention to include a quotation from every participant.

**Table III.** Example of child and parent perspectives within a subtheme.

Subtheme	Subcategory	Children´ s perspective	Parents´ perspective	Illustrative excerpts
Gaining motivation for and competence in physical activity	Experiencing increased physical competence and pride	Children expressed happiness and pride in their newly developed physical abilities and achievements.	Parents described increased physical competence as a source of pride and satisfaction for their children.	**Child:** “Yes, I have my own swimming card!” **Parent:** “Now he can swim. And I am very proud and impressed by that.”

**Table IV.** Subcategories, subtheme and theme.

Subcategories	Subthemes	Theme
Getting help with tailoring movements for the first time Enabling access to physical activities despite financial barriers Enabling participation despite disabilities Integrating participation despite cultural, geographical and age-related barriers	Feeling grateful when given access to physical activities	A door opener to physical capability and social community on one’s own terms
Experiencing increased physical competence and pride Gaining motivation through structure and technical aids	Gaining motivation for and competence in physical activity	
Engaging in enjoyable and tailored physical activities Strengthening social interaction and community	Learning fun physical activities and making social connections	
Improving access to information about PAP Expanding activities and extending the PAP period Creating opportunities for peer interaction and community	Optimising PAP for the future	

The first author (EW), who conducted all interviews and had primary responsibility for the analysis, is a physiotherapist and was a doctoral student at the time of the study. EW had no previous professional involvement in PAP or prior contact with the participating schools, school nurses, or participants. Her professional background as a physiotherapist and the study’s salutogenic theoretical perspective informed her perspective on physical activity and health-promoting resources. These perspectives were considered throughout data collection and analysis, and interpretations were discussed with the co-authors as part of the triangulation process. The co-authors contributed complementary perspectives from physiotherapy, physical education, sports science, and youth health.

## Ethics

Ethical approval for the study was granted by the Swedish Ethical Review Authority (Dnr: 2019–02295, 2020–07108 and 2022-06577-02). Informed consent was obtained from all participants, including the parents of participating children under the age of 15 years. The participants were assured confidentiality and were informed of their right to withdraw from the study at any time.

## Results: a door opener to physical capability and social community on one’s own terms

The analysis resulted in one overarching theme: ‘a door opener to physical capability and social community on one’s own terms’. The theme captures both the children’s and the parents’ various experiences of PAP as a positive incentive to move and encompasses the four sub-themes ‘feeling grateful when given access to physical activities’, ‘gaining motivation for and competence in physical activity’, ‘learning fun physical activities and making social connections’, and ‘optimising PAP for the future’.

The children and parents gave slightly different reasons for engaging in PAP. The children primarily said they found the prescribed activities fun, and the adolescents said they wanted to increase their social interactions and reduce physical inactivity. Meanwhile, the parents mentioned combatting overweight, facilitating access despite lack of economic means, and having a chance to develop physical skills as reasons. Although experiences of PAP were predominantly positive, participants also described limitations and unmet needs related to access to activities, affordability, the duration of PAP, and the extent to which PAP was needed. These differences were retained in the analysis and are reflected throughout the sub-themes, while the overarching theme captures experiences shared across the two groups.

Employing Bandura (1977), engagement in PAP appears to strengthens children’s self-efficacy in relation to physical activity; in addition, it fosters social community, in line with Antonovsky’s theory on overall sense of coherence (1979). Following McCuaig and Quennerstedt (2018) salutogenic approach, the sub-themes can be interpreted as reflecting the enhancement of various health resources that open a door to physical capability, fostering a sense of social belonging on the children’s own terms, providing equity in access to physical activity, and maintaining a sense of coherence through the sociocultural dimension.

### Feeling grateful when given access to physical activities

The first sub-theme, ‘feeling grateful when given access to physical activities’, indicates that engagement in PAP strengthens health resources from a salutogenic perspective. That is, gratitude for inclusion in physical activities enhances children’s sense of coherence and sense of being valued despite coming from a somewhat marginalised or subordinated position.

From the parents’ perspective, PAP addressed a need for support in enabling their children to become more physically active on their own terms and according to their individual prerequisites. The parents, who generally described previous experiences of physical activity themselves, expressed particular appreciation when their children engaged in PAP. Furthermore, the parents appreciated how PAP helped their children feel included in physical activities.

Emma: I was shocked when the school nurse mentioned PAP. It had not been in my mind. I think it’s a very good project, especially given today’s society; there isn’t much activity for children. So that I think it has been very positive. I really hope it’s something that will continue for children in the future.

Further, the parents highlighted that the PAP intervention was the first time they received help in tailoring physical activities for their children. Before engaging in PAP, they had only received general information about the importance of physical activity. For example, one father described disappointment about health care’s narrow focus on BMI and weight reduction.

Anders: We had to solve the problem with weight the best we could. The only thing we got at the Children’s Healthcare Centre was that he had to lose weight. Those words. The first contact with the nurse, she told me straight to my face: ‘he is fat!’ So, it was completely wrong behaviour from her.

From the adolescents´ perspective, PAP was appreciated as an opportunity to remain physically active at an age when they perceived that many peers stopped being physically active or participating in organised sports. At the same time, they also expressed needing support at an earlier age. Similarly, a father to a boy who recently started school wanted his son to have support with physical activity early, at the age of four or five, because there was almost no support for physical activity then.

Affording physical activity was raised as an issue by the participants, which highlights how PAP was viewed as subsidising costs for physical activities. According to the parents, PAP made it more manageable to let their child try an activity because they did not have to cover the entire cost themselves. They mentioned that sport activities were often expensive, but PAP gave children the opportunity to try an activity to see if it suited them before parents with limited finances paid money out of their own pockets.

Emma: I’m a single mom, so spending money on swimming several times a week has been a concern. Having the subsidised cost of swimming through PAP has therefore been much appreciated.

However, PAP did not remove all financial barriers to physical activity. Some activities that children found enjoyable required equipment or costs that could not be subsidised through PAP, such as horse riding or cycling.

The first author: We can check the pictures here. Is there anything that you find fun here?

[Amir points to the picture with a bicycle.]

The first author: To ride a bike. Do you usually cycle to school?

Amir: No, I don’t have a bike. [The mother later confirms that they cannot afford to buy a bike.]

Parents of children with neuropsychiatric disorders described PAP as helping their children find activities that were compatible with their individual needs. They had previously struggled with finding suitable activities for their children because diagnoses like ADHD and autism created difficulties with concentration and social interaction. Emma, the mother of a boy with a neuropsychiatric disorder, explained,

Emma: He has problems with team sports. He himself felt that it became boring, but I, as a parent, saw that it is because he has problems playing as a team. So, there it was a bit problematic. But individual activities, like at the trampoline centre, he thought it was great fun. Then he could focus only on himself.

This illustrates that participation in physical activity was not experienced as equally accessible across activity contexts, and that an individualised choice of activity was important for some children.

This first theme also captures how PAP helped children integrate into physical activities despite obstacles related to cultural background and geography. It points to perceived inclusion despite previous challenges of exclusion. From the child´ s perspective, PAP could also facilitate entry into an unfamiliar sports context. For instance, Rayan, a boy who had lived in Sweden for two years, felt PAP helped him integrate into Swedish sports culture. His parents, who fled from a non-European country, had no knowledge of Swedish sports clubs. With support from his school nurse, who prescribed football, he joined a football club.

Rayan: Many friends and teachers told me, ‘Rayan, you are good at football, why aren’t you in a club?’ One day I realised that I wanted to join a football club, but I needed help to get in. I got help with everything via PAP.

The first author: Okay, did you get help contacting any football clubs? Did the school nurse do it?

Ryan: Yes, she did.

From a salutogenic perspective, this theme illustrates the mobilisation of health resources such as inclusion, sense of coherence, and value. Based on Antonovsky (1979) sense of coherence concept, it shows that PAP makes physical activity manageable and meaningful. The participants also raised vital aspects on equity in physical activity, underscoring how PAP facilitated access to physical activities despite perceived barriers such as economy, disabilities, and geographical location.

### Gaining motivation for and competence in physical activity

The second sub-theme, ‘gaining motivation for and competence in physical activity’, reflects experiences of pride in new activities. The theme captures the sense of capability and increased physical activity gained from two health resources attributed to PAP: physical competence and intrinsic motivation for movement. Parents mainly described changes they observed in their children, such as increased activity, motivation, and physical competence. The children themselves described these changes in terms of happiness, pride, and a sense of capability.

The parents emphasised that the prescription contributed to a higher level of physical activity, which strengthened their children’s motivation and physical competence. One mother highlighted that PAP had motivated her son to go to the swimming pool, and now he can swim.

Emma: He starts sixth grade in the fall, and they must be able to swim to get a pass in PE. He couldn’t swim, and I mentioned that to the school nurse, and then she suggested a physical activity prescription, so we could go to the swimming pool and train swimming. I thought that sounded great, so now we have been there for two months. Now he can swim. And I am very proud and impressed by that.

Johan, whose daughter had completed the prescription, said that she was ‘proud and happy’ over what she had accomplished during PAP. The children themselves also expressed happiness and pride in their new physical capacity, as well as the access to physical activities.

The first author: Your mother told me that you liked going to the swimming pool.

Amir [smiling proudly]: Yes, I have my own swimming card!

The prescription was perceived to contribute to motivation for more physical activities, by both the children and the parents. Amanda, a teenage girl, said that before PAP, she had a desire to become more physically active but lacked motivation. a reason to become physically active and that gave motivation.

Amanda: The good thing about PAP is that I now have a reason to go out and train once a week. I get kind of motivated by that.

One father expressed that the structure of PAP increased his son’s motivation to be active, and now the son had started to suggest physical activities by himself, such as playgrounds and swimming pool visits. According to Noura, whose son received PAP, the prescription was structured and had ‘opened doors’ for more physical activities. She also saw it as a tool that could continue to support her son in the future.

Noura: PAP is great at helping the children and creating opportunities. PAP supports the family to help the children to be physically active. When the prescription is over, we parents take care of this. The children cannot have the prescription for one, two, or three years. PAP opens possibilities for the children and teaches children to be physically active and then they want to continue with that activity.

Furthermore, Maja, a 12-year-old girl, described how the opportunity to borrow technical training aids (a pedometer) from the school nurse during the prescription period had increased her motivation for physical activity. Her father confirmed that the pedometer motivated her to be more active, and after the prescription, he bought her one.

Johan: She checked how many steps she took and wrote it down. Next day, she wanted to try to take more steps. It became her own little competition to get as many steps as possible. She makes things up that she could get steps for, TikTok and stuff.

Overall, this sub-theme illustrates how PAP mobilised health resources related to personal well-being, feelings of pride, motivation to move, and a sense of capability among the children. It also captures how both the children and their parents gained a ‘motivational boost’ to move, viewing movement as meaningful. Regarding sense of coherence (Antonovsky, 1979), engaging in PAP seemed to mobilise the health resources of personal well-being as emotional sense-making, creating both meaningfulness and manageability.

### Learning fun physical activities and making social connections

The third sub-theme shows the mobilisation of several health resources. PAP enables children to engage in enjoyable physical activities, which can be considered a health resource that enhances their sense of meaningfulness. Moreover, both children and parents valued the social interactions that these activities entailed, which appear vital for building strong relationships.

When talking about their PAP activities, the children mainly expressed positive experiences, using words such as ‘very fun’ and giving thumbs up. They pointed out that they had chosen their activity themselves, so they were able to do something that they thought was fun. Charlie said that he and his mother went to the swimming pool on a certain day of the week because there was an obstacle course in the water that day, which he liked, whereas Amir said that he had chosen swimming because there was a water slide that he thought was fun.

The first author: I’ve never been to the swimming pool here in town. Are there many things to do?

Amir: Yes, there is. The red slide is the most fun. It is the fastest slide.

The parents also stressed the importance of the PAP activity being fun for the child, for example, expressing that ‘when the child doesn’t think the activity is fun, then nothing happens’. Therefore, they said it was important that the child was allowed to participate in deciding the activity, so that the activity was experienced as joyful rather than ‘a compulsion’. According to the parents, tailored PAP meant that the child had the opportunity to get help in finding an activity that they liked.

The first author: Are you satisfied with football as a PAP activity for your child?

Malika: Yes! Because it is his dream to become a football player.

Thus, while the children primarily described enjoyment as an immediate experience of the activity, parents emphasised the importance of ensuring that the activity was enjoyable in order for their child to engage in it.

The youngest children (and their parents) named the specific activities, such as swimming or walking, instead of saying ‘prescription’. They explained that they did so to make the activities more understandable and enjoyable for the child.

Moreover, enriching social interactions and building communities with other children during the PAP activities contributed to the activities being experienced as fun, according to the children. Amir, whose prescription was swimming, said that he thought it was joyful because he brought his siblings with him and, sometimes, he met friends at the pool. In addition, the prescription helped children to make new friends, even outside of school.

The first author: How did it feel when you went to football practice?

Rayan: It was great fun! A lot of my friends from school were there in the club.

But I also got to know a lot of new people.

Amanda: I don’t meet friends that much in everyday life. I only meet people at school, so it’s nice to have a reason to meet friends outside of school. [Amanda received PAP together with a classmate.]

The opportunity to involve friends and family members in the activity was also emphasised by parents, and some parents considered the social interactions as the most important reason for their child doing PAP. For example, Karim said that it was an eye-opener for everyone in the family that they all need to be more physically active: thanks to the child’s prescription, the whole family became more physically active.

For the children, social interaction was described as part of what made the activity enjoyable and meaningful. For some parents, however, the social dimension was viewed more broadly as an important reason for supporting their child’s participation.

In summary, this sub-theme reveals health resources that can create ‘meaningfulness’ in relation to Antonovsky (1979) sense of coherence theory.

The (physical) activities seemed meaningful when they brought social interactions with others, when the children were involved in the decision-making process, and when they were perceived as fun. This suggests that when children get opportunities to influence the prescribed activity, they find it ‘manageable’.

### Optimising PAP for the future

The final sub-theme, ‘optimising PAP for the future’, comprises suggestions for enhancing PAP. These suggestions have the potential to further strengthen salutogenic health resources by fostering social community and providing tailored and enjoyable activities.

Several children were interested in creating a social community and interacting with other children who received PAP. They explained that communicating with other children was a chance to exchange ideas and increase motivation for physical activity. According to Maja, 12 years old, communication could take place via social platforms such as Facebook, messenger, or TikTok. Hence, the children saw social media as a way to organise activities.

The parents also wanted to build a community to exchange ideas. They expressed different thoughts about the format, including communication on digital platforms.

Johan: Maybe there were several children in school who had PAP and could have a PAP club. But it doesn’t have to be at school; it can also be a contact group for children around Sweden, where the children can discuss or make TikToks together. So that you get a community.

The children and parents also differed somewhat in how they described future development of PAP. The children primarily suggested opportunities for peer interaction and social community, whereas parents more often focused on the structure, duration, affordability, and targeting of the intervention.

All the interviewed children were satisfied with their PAP activity, but they expressed a need for more activities, such as horse riding, gym, and trampoline jumping, to suit everyone. Further, some parents pointed out that the PAP timeframe (three months) needed to be extended to at least six months, as it took time to get into the activity and create a habit.

Karim: It would have been good if you could extend PAP to six months, so the child really gets into it. Now during the wintertime, there are a lot of illnesses and colds. Three months go by fast, so you need a little more time.

Moreover, the parents suggested that the information about PAP could be improved to reach children who need it most. However, some parents pointed out the risk in giving information to everyone, because everyone might want PAP. They suggested that the focus should instead be on informing those with the greatest need. In contrast to the parents’ statements, a teenager named Sofia reported that the information about PAP was given in the classroom to all children, which is how she got interested. Importantly, Sofia described herself as already physically active and questioned whether she needed PAP to increase her physically activity. Instead, her main motivation was opportunity to engage in a physical activity with friends. She said:

Sofia: I probably didn’t need any PAP, because I do movements a lot anyway. But swimming with my friend is fun, so I still wanted to do it.

Sofia’s experience contrasts with the experiences of participants who described PAP as an important means of initiating or increasing physical activity, and highlights that PAP could also be valued for social rather than primarily physical activity-related reasons.

In summary, the final sub-theme also reflects a salutogenic perspective incorporating sociocultural dimensions (Antonovsky, 1979). It suggests optimising PAP to further enhance health resources, thereby increasing the meaningfulness of using PAP. Both children and parents suggested the use of social media and digital solutions to improve communication and build community around physical activities.

## Discussion

Our results indicate that children who received Physical Activity on Prescription (PAP) in the school setting, together with their parents, perceived the programme as a ‘door opener’ and as a catalyst for experiences of physical capability and social belonging on the children’s own terms. This perspective contrasts with earlier research involving professionals, which described the PAP process as more complex and ‘delicate’ (Wiklund et al., 2025).

The results reveal a marked discrepancy between professionals’ and users’ perceptions of physical activity on prescription. While school nurses and physical education teachers express concerns that the intervention may be difficult to implement and potentially stigmatising (Wiklund et al., 2025), children and parents instead describe it as supportive and much needed. Parents emphasise a sense of relief and gratitude that someone finally acknowledges their situation and offers concrete help. This contrast suggests a gap between a professional logic characterised by caution and concern about unintended negative effects, and a user perspective grounded in experienced need and appreciation of support. In this sample, children and parents generally described the intervention as a form of recognition and care rather than as stigmatising. These findings highlight the importance of incorporating user perspectives in both the design and implementation of health interventions. However, the absence of reported stigma in our sample should not be interpreted as evidence that stigma is uncommon or that professionals’ concerns are unfounded. Children and families who experienced stigma, declined PAP, discontinued participation, or had less favourable experiences may have been less likely to be identified through the recruitment process or to participate in the study. Thus, the present findings complement rather than contradict previous research highlighting professionals’ concerns about stigma.

The predominantly positive experiences reported by participants should nevertheless be interpreted in light of the study context and recruitment procedure. As participation was mediated by school nurses, families who were more engaged with PAP or who had more positive experiences may have been more likely to participate. In addition, the study included children and parents whose PAP was ongoing or had been completed within the previous year, which may have influenced the salience of experiences related to the intervention. The study therefore cannot determine whether the predominantly positive accounts are representative of families with less favourable experiences or those who did not participate.

In line with these user experiences, children and parents in our study articulated perspectives that align with a salutogenic and non-medical orientation (Quennerstedt, 2008), emphasising the value of sociocultural integration. By conceptualising these experiences as ‘health resources’ through a salutogenic health lens (McCuaig & Quennerstedt, 2018), we suggest that engaging in PAP mobilised personal well-being and social interaction, increased motivation, and facilitated engaging in enjoyable physical activities. This contribution is particularly valuable because existing literature on school-based physical activity interventions has been problematized as medically driven, focusing primarily on quantifying health outcomes (Andermo et al., 2025; Larsson & Thedin Jakobsson, 2023).

As our results show, the mobilisation of health resources helps individuals to perceive life as *comprehensible*, *manageable*, and *meaningful* (Eriksson & Lindström, 2006), according to Antonovsky, 1987, 1996) sense of coherence theory. Both comprehensibility and manageability are evident in the parents’ and children’s appreciation of the structured and individualised nature of PAP, which creates support for the family. PAP activities seem manageable when the children are involved in the decision-making process, and the individualised structure contributes to a sense of inclusion regardless of socioeconomic disadvantages, disability, and cultural background. Previous research criticised individual physical activity interventions in schools for running the risk of missing the context and laying the responsibility of participating in physical activity on children (Karlander & Geidne, 2025; Larsson & Thedin Jakobsson, 2023). However, this study shows that PAP, despite being individualised, takes the social context into account and gives support and structure to families who felt excluded in previous movement contexts (organised sports or the school subject PE), which can be interpreted as feelings of stigmatisation. The family’s inclusion in PAP can be interpreted as a shift of power from SHS to the family. Historically, SHS has focused on remedial medical interventions, such as weight loss, with little consideration for the child’s perspective (Holmström et al., 2015). PAP provides children with structure and support while allowing them to act and take responsibility for themselves. Based on Bandura (1977) theory of self-efficacy, participants’ accounts can be interpreted as reflecting increased confidence and perceived capability in relation to physical activity.

Participants’ accounts suggest that PAP was associated with experiences that can be interpreted as involving several health resources related to Antonovsky, 1996) key concept of *meaningfulness*. They show how engaging in enjoyable physical activities is meaningful to children, which is in line with Geidne and Quennerstedt (2021). This is also supported by Tay et al. (2021) showing that enjoyment and having fun are among the strongest motivators for children´ s engagement in physical activity, often outweighing awareness of long-term health benefits. Nevertheless, the concept of enjoyment can vary widely (Skille & Østerås, 2021), and the meaning of fun and enjoyment in sports is complex (Persson et al., 2020). For example, Thedin Jakobsson and Lundvall (2021) understand children’s participation in sports because it is fun as a sense of meaningfulness that requires a certain ‘habitus’ with a taste for sports (Thedin Jakobsson, 2015). However, in this study, the children lack the habitus for sport, but they still describe positive experiences of movement initiated by PAP. This may be attributed to PAP offering children the opportunity to discover their driving forces, intrinsic motivations, and health resources through physical activities that were not necessarily associated with traditional sports or dependent on a specific sports habitus. This interpretation is further supported by the children’s suggestions for more PAP activities facilitated by commercial providers, such as gyms, trampoline parks, and similar venues. These preferences align with previous research indicating that such forms of activity are perceived as meaningful by children and can foster sustained engagement over time (Högman, 2021). Moreover, several participating children had neurodevelopmental disorder diagnoses, which often create challenges in accessing and participating in organised sports (Geidne & Jerlinder, 2019). This highlights the potential of PAP to offer inclusive and adaptable alternatives.

Further, we argue that the enjoyable self-selected activities and the individualised structure of PAP contribute to a ‘motivational boost’ for movement, making physical activity meaningful for the children. PAP was perceived as a motivator for movement beyond just health benefits, which often concern pathogenic causes and disease risks. In contrast, Högman (2024) found that children receiving PAP from a health clinic viewed physical activity primarily in terms of health benefits rather than seeing it as a source of intrinsic motivation. This implies that children’s experiences of PAP may vary depending on whether the prescription is given in a salutogenic school context or a more pathogenic healthcare context.

Additional aspects that contribute to meaningfulness include PAP being experienced as facilitating social interactions with family and friends, giving meaning to the activity, which aligns with previous research (Hesketh et al., 2017; Maric et al., 2020). The participants also suggested optimising PAP to support their sense of social belonging and thereby meaningfulness by using social media and digital solutions to improve communication and community around physical activities. This can strengthen salutogenic health resources such as fostering a digital social community. That children suggested boosting PAP and digital communities with social media is hardly surprising, as social media plays a central role in their lives (Public Health Agency of Sweden, 2024). Research has identified both pros and cons in using social media to promote physical activity (Goodyear & Armour., 2019; Goodyear et al., 2021., 2021), and Quennerstedt (2019a) discussed social media as a health resource with the potential to be a tool for social relations.

Although we identified health resources to conceptualise the results from a salutogenic perspective, there are perspectives that a narrow salutogenic conceptualisation does not capture (Eriksson, 2015; Quennerstedt, 2019b). Therefore, the integration of social and sociocultural perspectives adds important insights to the experiences of PAP ‘on one’s own terms’. For example, the perspective of equity in physical activity promotion is reflected in some parents talk about the risks of offering PAP to everyone instead of those with the greatest need.

Previous research has underscored the role of school nurses in the PAP process while also noting its time-consuming nature and lack of clear guidelines (Wiklund et al., 2025). These findings raise questions about how PAP should be targeted and which children may benefit most from the intervention. Some parents in our study suggested that information about PAP should primarily reach children with the greatest need, whereas one adolescent participant described herself as already physically active and questioned whether PAP was needed to increase her physical activity. Instead, she valued the opportunity to engage in a physical activity with a friend. This contrast suggests that perceived need for PAP may not always correspond to physical activity level, as social opportunities and access to enjoyable activities may also motivate participation. At the same time, given the potential selection bias in our sample, these findings should not be interpreted as evidence that PAP should be offered universally or that all children who express interest would benefit from it. Rather, PAP may represent one component within a broader palette of health-promoting strategies for children, with the relevance of the intervention depending on individual needs and circumstances.

Importantly, PAP in the school context remains an underexplored area. Further research is needed to assess its feasibility and acceptability in educational settings. Future studies could build on the outcomes identified in this study—such as the increased motivation for movement, enhanced physical competence, strengthened sense of community, and enhanced well-being—rather than focusing solely on traditional health metrics. It would also be valuable to investigate whether additional health resources, such as physical self-esteem, can be improved through PAP.

## Strengths and limitations

The qualitative approach, through individual interviews and qualitative content analysis (Graneheim & Lundman, 2004), captured children’s and parents’ own experiences of PAP. A strength of the research process was the attention to trustworthiness by enhancing credibility, dependability, confirmability, and transferability (Graneheim & Lundman, 2004; Graneheim et al., 2017). To enhance *credibility*, we selected participants and schools to represent diverse demographics, including age, gender, ethnicity, socioeconomic status, and urban/rural settings. Data triangulation was employed among the authors to achieve analytical richness and consensus. The varied expertise of the authors further strengthened the credibility. *Dependability* was ensured by having the same author conduct all interviews using a semi-structured guide. The authors’ extensive experience in conducting research with children of various ages and disabilities, including the ability to adapt the interview process with image support, was a significant strength. *Confirmability* was ensured through multiple readings and research team discussions. The participant quotations in the results illustrate the rich content and enable readers to assess the confirmability of the findings. Participant validation (member checking) was not undertaken. Instead, credibility was supported through investigator triangulation, repeated discussions within the research team, and transparent presentation of participant quotations. Although participant validation may have provided additional perspectives on the interpretation of the findings, it was not part of the study.

The study also has limitations, including the relatively small (albeit diverse) sample and potential selection bias. The heterogeneity of the sample, including variation in age, ethnic background, and the presence of autism or other mental health conditions among some children, may also have influenced the findings. These characteristics may shape children’s and parents’ experiences of PAP in different ways, and the small sample did not allow us to examine such differences systematically. The diversity of the sample may therefore have contributed to a broader range of experiences being represented, while also making it difficult to determine whether particular experiences were related to PAP or to individual or contextual characteristics.

No predefined sample-adequacy criteria, such as information power or data saturation, were applied. Instead, recruitment aimed to achieve variation in participants characteristics and continued until the research team considered the data sufficient to address the study aim. Recruitment relied on professional gatekeepers (e.g., managers and school nurses), which may have contributed to selection bias if families with more positive experiences of PAP were more likely to be approached or to accept participation (Johansson & Karlsson, 2013). Consequently, children who declined PAP, discontinued participation, or had less favourable experiences may not be represented in this study. The findings should therefore be interpreted as reflecting the experiences of participating children and parents rather than all children offered PAP. In addition, participants may have been inclined to present themselves in a favourable light or emphasise positive experiences of physical activity and PAP, resulting in socially desirable responses. Such bias may have reduced the reporting of challenges or negative experiences.

Further, the authors’ generally positive orientation toward physical activity and PAP may have influenced both the interpretation of the data and the framing of the findings. Throughout the analysis, reflexive discussions were held within the research team, and alternative interpretations were actively considered. The integration of theoretical perspectives problematizing assumptions regarding physical activity and PAP (Wiklund and Vikman, 2024a, 2025, Wiklund et al., 2024b) further supported a critical interpretation of the findings. Nevertheless, the influence of the researchers´ pre-understanding cannot be fully excluded.

Most child interviews were conducted with a parent present. While this may have increased children’s comfort and facilitated participation, it may also have influenced what children chose to disclose or encouraged socially desirable responses. Likewise, including interviews from both children and their parents provided complementary perspectives on the PAP experience but may also have reinforced shared family narratives. Furthermore, the study design did not allow us to distinguish experiences specifically attributable to PAP from those related to family support, school initiatives, or other concurrent experiences. Similarly, the study was not designed to assess adherence to PAP or objectively determine changes in physical activity. Participants’ accounts of increased activity and motivation should therefore be understood as perceived experiences rather than evidence of adherence or intervention effects. The findings should therefore be interpreted as participants´ experiences of PAP within their broader everyday context rather than as effects of PAP alone.

The interviews were conducted both digitally and in person. The digital format facilitated recruitment and participation (Edwards & Holland, 2020), but it may have affected spontaneity and depth. Further, the absence of body language may have influenced dialogue interpretation (Edwards & Holland, 2020; Lo Iacono et al., 2016). Method triangulation was beneficial for studying children’s views and experiences (Emm-Collison et al., 2022). Allowing participants to choose their preferred format may have increased comfort, potentially reducing interviewer effects (Thunberg & Arnell, 2022).

The specific Swedish school context may have limited the transferability of results to international contexts due to differences in SHS and physical activity opportunities. However, analytical transferability is possible (Graneheim & Lundman, 2004).

## Conclusion

The results indicate that children and parents see PAP as a door opener to improving physical capability and fostering a sense of social belonging on their own terms. At the same time, they suggest ways to optimise PAP by building community through digital technologies, providing more tailored and enjoyable physical activities. Participants described the structured and individualised nature of PAP as supporting motivation and physical competence, which contributed to making physical activity more enjoyable. The children and parents talked about PAP in terms that can be interpreted from a salutogenic perspective, conceptualised as ‘health resources’ permeated by sociocultural dimensions. These findings reflect participants’ experiences and should not be interpreted as evidence of intervention effects or long-term changes in physical activity.

## Data Availability

The data are not publicly available due to containing information that could compromise the privacy of research participants. Not sharing the analysed data openly follows the current legislation provided by the ethical review board.
